# Development and validation of an algorithm to recalibrate mental models and reduce diagnostic errors associated with catheter-associated bacteriuria

**DOI:** 10.1186/1472-6947-13-48

**Published:** 2013-04-15

**Authors:** Barbara W Trautner, Rupal D Bhimani, Amber B Amspoker, Sylvia J Hysong, Armandina Garza, P Adam Kelly, Velma L Payne, Aanand D Naik

**Affiliations:** 1 Houston Health Services Research and Development Center of Excellence, Michael E. DeBakey VA Medical Center, Houston, TX, USA; 2 Department of Internal Medicine, Baylor College of Medicine, Houston, TX, USA; 3 Department of Internal Medicine, Tulane University School of Medicine, New Orleans, LA, USA; 4 Michael E. DeBakey VA Medical Center (152), 2002 Holcombe Boulevard, Houston, TX 77030, USA

**Keywords:** Catheter-associated bacteriuria, Urinary tract infections, Evidence based guidelines, Diagnostic errors

## Abstract

**Background:**

Overtreatment of catheter-associated bacteriuria is a quality and safety problem, despite the availability of evidence-based guidelines. Little is known about how guidelines-based knowledge is integrated into clinicians’ mental models for diagnosing catheter-associated urinary tract infection (CA-UTI). The objectives of this research were to better understand clinicians’ mental models for CA-UTI, and to develop and validate an algorithm to improve diagnostic accuracy for CA-UTI.

**Methods:**

We conducted two phases of this research project. In phase one, 10 clinicians assessed and diagnosed four patient cases of catheter associated bacteriuria (n= 40 total cases). We assessed the clinical cues used when diagnosing these cases to determine if the mental models were IDSA guideline compliant. In phase two, we developed a diagnostic algorithm derived from the IDSA guidelines. IDSA guideline authors and non-expert clinicians evaluated the algorithm for content and face validity. In order to determine if diagnostic accuracy improved using the algorithm, we had experts and non-experts diagnose 71 cases of bacteriuria.

**Results:**

Only 21 (53%) diagnoses made by clinicians without the algorithm were guidelines-concordant with fair inter-rater reliability between clinicians (Fleiss’ kappa = 0.35, 95% Confidence Intervals (CIs) = 0.21 and 0.50). Evidence suggests that clinicians’ mental models are inappropriately constructed in that clinicians endorsed guidelines-discordant cues as influential in their decision-making: pyuria, systemic leukocytosis, organism type and number, weakness, and elderly or frail patient. Using the algorithm, inter-rater reliability between the expert and each non-expert was substantial (Cohen’s kappa = 0.72, 95% CIs = 0.52 and 0.93 between the expert and non-expert #1 and 0.80, 95% CIs = 0.61 and 0.99 between the expert and non-expert #2).

**Conclusions:**

Diagnostic errors occur when clinicians’ mental models for catheter-associated bacteriuria include cues that are guidelines-discordant for CA-UTI. The understanding we gained of clinicians’ mental models, especially diagnostic errors, and the algorithm developed to address these errors will inform interventions to improve the accuracy and reliability of CA-UTI diagnoses.

## Background

Catheter-associated urinary tract infection (CA-UTI) and catheter-associated asymptomatic bacteriuria (CA-ABU) are very common yet distinct forms of catheter-associated bacteriuria [1,2]. In CA-UTI, the patient has specific urinary symptoms, and the condition merits treatment with antibiotics [1]. In contrast, CA-ABU is marked by the absence of urinary-specific symptoms, and treatment with antibiotics does not reduce mortality, bacteremia, or subsequent risk of UTI [2]. Thus, both the Infectious Diseases Society of America (IDSA) and US Preventive Services Task Force discourage screening for, and treatment of, CA-ABU in most clinical settings [3]. Recent guidelines by IDSA provide excellent summaries of the evidence supporting these recommendations [1,2].

Despite the IDSA guidelines, inappropriate treatment of CA-ABU with antibiotics is widespread, and guidelines adoption remains modest [4]. Recent studies of CA-ABU in hospital settings show as many as 52% of patients with CA-ABU being treated unnecessarily with antibiotics [5-9]. The cause of CA-ABU overtreatment is multifaceted and grounded in the clinical norms and inappropriately constructed mental models clinicians use to make diagnostic decisions for patients with catheter-associated bacteriuria. Conventional teaching is that the bladder and the urine within it are sterile, but this “norm” does not apply to catheterized patients in contemporary medical settings. Making the diagnosis of CA-ABU requires the clinician to discount clinical cues, such as bacteriuria and pyuria, because neither of these can be used to distinguish between CA-ABU and CA-UTI [1,2,10]. Another clinical norm that runs counter to evidence is the erroneous belief that vague, non-urinary symptoms can be attributed to bacteriuria [11-14]. Additionally, clinicians often overweigh the risk of withholding antibiotics while underweighting the risk of antibiotic exposure in an individual patient [15]. These evidence-discordant norms and biases produce decision-making processes that differ in distinct and clinically important ways from evidence-based guidelines for diagnosing catheter-associated bacteriuria [16,17].

The IDSA guidelines governing catheter-associated bacteriuria are based on high-quality reviews of the available evidence [1,2]. However, the complexity and sheer length of the guidelines (51 pages) may impede their uptake [18,19]. Classically, diagnostic reasoning is thought to involve complex, analytical evaluations of clinical and laboratory cues to frame prior probabilities of differential diagnoses to arrive at the accurate diagnosis [20]. Empiric evidence suggests that clinicians store disease models reflecting common symptoms associated with diseases within their memory [21]; these models are called ‘mental models’. These mental models are normally constructed during training when clinicians learn the symptoms associated with diseases, and are enhanced as they gain experience throughout their career. When the mental models are not accurate, diagnostic errors may occur. Use of inaccurate (guidelines-discordant) mental models associated with CA-ABU can result in misdiagnosing CA-ABU as CA-UTI. Mental models for CA-UTI that are properly constructed (guidelines-concordant) are reflected in Table 1 left column. A guidelines-discordant mental model for CA-UTI (commonly used when misdiagnosing CA-ABU as CA-UTI) is shown in the right column of Table 1.

**Table 1:** Components of Clinicians’ Mental Models Diagnosis of Catheter-Associated Urinary Tract Infection (CAUTI)

**Guideline concordant signs and symptoms of CAUTI**	**Guideline discordant signs and symptoms of CAUTI**
Fever	Pyuria (white blood cells in urine)
Delirium	Foul smelling urine
Rigors	Change in urine color
Flank pain	Sediment in urine
Acute hematuria (red blood cells in urine)	Systemic leukocytosis (higher than normal white blood cell count)
Pelvic discomfort	Prior “UTI” diagnosis
Urgency	Resistant organism in urine
Frequency	Vague malaise
Dysuria	Weakness
Suprapubic pain	Type of organism in urine

In essence, prior research demonstrates that inappropriate treatment of CA-ABU with antibiotics is widespread and guidelines adoption remains modest. To address this problem, the first objective of this research was to confirm our suspicion that clinicians’ mental models are inaccurately constructed and to find the points of difference from evidence-based guidelines. The second objective was to develop a means of re-directing clinicians’ mental models by creating a valid and reliable algorithm grounded in clinical evidence, with the ultimate objective of informing a guidelines implementation intervention.

## Methods

We framed the problem and our approach to the problems using a two phase study approach based on the following hypotheses. First, when clinicians attempt to differentiate catheter-associated bacteriuria as either CA-UTI or CA-ABU, their mental models include both guidelines-discordant and guidelines-concordant cues resulting in (a) poor diagnostic accuracy differentiating between CA-UTI and CA-ABU (reliability with clinical guidelines and/or clinical experts) and (b) low rates of diagnostic agreement between each other (low inter-rater reliability among non-experts). Phase 1 evaluated the accuracy and inter-rater reliability of clinicians’ mental models for catheter-associated bacteriuria. Second, we distilled the IDSA guidelines into an algorithm to attempt to improve diagnostic accuracy and inter-rater reliability by substituting guidelines-concordant cues in place of guidelines-discordant cues. In essence, the algorithm serves to recalibrate clinicians’ mental models for differentiating CA-UTI from CA-ABU. Phase 2 describes the development, preliminary validation, and evaluation of inter-rater reliability of this algorithm for recalibrating clinicians’ mental models. This research was conducted with the approval of the Baylor College of Medicine Internal Review Board (protocol H #24180).

### Phase 1 – Clinicians’ decision-making when diagnosing CA-UTI

#### Study design

We conducted a comprehensive assessment of the diagnostic cues clinicians use when distinguishing between CA-UTI and CA-ABU, through a case-based diagnosis exercise followed by in-depth, cognitive interviews [22].

#### Participants

Participants consisted of six physicians and four allied health professionals recruited from a convenience sample of experienced clinicians working in local acute and extended care facilities of a single health system. The sample of clinicians included three males and seven females with a range of 11-15 years of experience treating older patients in long-term care and inpatient settings (see Table 2). All 10 participants reviewed each of the four cases for a total of 40 cases on which analyses are based.

**Table 2:** Characteristics of study participants

	**Phase 1 participants (n=10)**	**Phase 2 participants (n=6)**
**Characteristic**	**Number (%)**	**Number (%)**
Female gender	7 (70)	4 (67%)
Occupation		
Physician	6 (60)	4 (67%)
Physician’s Assistant	2 (20)	1 (17%)
Nurse Practitioner	2 (20)	1 (17%)
Duration of Clinical Experience		
1-5 years	1 (10)	4 (67%)
6-10 years	3 (30)	
11-15 years	5 (50)	2 (33%)
>15 years	1 (10)	

#### Procedures

Clinicians were asked to review the electronic medical records of four patients with positive urine cultures associated with an indwelling urinary catheter. Henceforth, for simplicity, we will refer to these positive urine cultures, both bacteriuria and funguria, as “bacteriuria”. All participants independently reviewed the same four urine cultures, representing four distinct cases. The selected cases were actual patient cases representing a spectrum of clinical cues and treatments representative of CA-UTI and CA-ABU. Each case presentation had at least one clinical cue shown in prior studies to influence physicians’ decision-making regarding antimicrobial treatment (e.g., older age, pyuria, and type of organism) [6,23]. Table 3 describes the patient cases. For each of the four cases, clinicians answered two written questions: (1) Do you feel this is a CA-UTI or CA-ABU? and (2)What helped you decide if this case was a CA-UTI or CA-ABU? Subjects then underwent a cognitive interviewing exercise [22] to elicit their reasoning processes where they answered the following question: “For the case of Patient X, was your decision of CA-UTI versus CA-ABU influenced by any of the following?” Choices included pyuria (white blood cells in urine), systemic leukocytosis (white blood cells in bloodstream), type of organism in the urine (Gram negative, Gram positive or fungal), elderly or frail patient, weakness, cloudy urine, foul-smelling urine, and specific urinary symptoms (e.g. dysuria—painful urination or frequency). The researcher stated each probe one-at-a-time, after which the clinician responded with a ‘yes’ or ‘no’. To assess use of the guidelines, clinicians were then asked “For the case of Patient X, do you feel that you applied the IDSA guidelines to arrive at your decision?” With participant consent, all interviews were tape recorded and subsequently transcribed and analyzed.

**Table 3:** Description of bacteriuria cases diagnosed by long-term care providers and signs/symptoms endorsed

**Case**	**Culture results**	**Diagnosis based on guideline criteria**	**Comments**	**Guideline-consistent diagnoses (%)**	**Guideline-concordant signs/symptoms endorsed (Number of clinicians)**	**Guidelines-discordant signs/symptoms endorsed (Number of clinicians)**
**1**	>10^5^ CFU/mL *Klebsiella Pneumoniae*	CA-ABU	Systemic leukocytosis, receiving systemic corticosteroids	6 (60%)	Urinary symptoms incorrectly identified as present (1)	Pyuria (5)
					Lack of fever (6)	Leukocytosis (7)
						Elderly/frail patient (5)
						Weakness (3)
						Organism number (1)
**2**	<10^4^ CFU/mL gram positive organisms	CA-UTI	Fever of 103.3 degrees, and no other source identified	4 (40%)	Lack of urinary symptoms (2)	Pyuria (3)
						Leukocytosis (4)
					Fever (6)	Organism type (3)
					Delirium (3)	Elderly/frail patient (5)
					Hematuria (3)	Isolated Organisms (3)
**3**	>10^5^ CFU/mL *E. coli;* and >10^3^ - <10^5^ CFU/mL *Klebsiella oxytoca*	CA-ABU	Leg weakness, no symptoms of urinary tract infection	1 (10%)		Pyuria (3)
						Leukocytosis (7)
						Organism type (6)
						Elderly/frail patient (5)
						Weakness (6)
						Patient fall (5)
						History of UTIs (3)
**4**	>10^5^ CFU/mL *Candida albicans*	CA-ABU	No symptoms of urinary tract infection	10 (100%)	Presence of respiratory symptoms [alternate cause] (4)	Lack of leukocytosis/mild Leukocytosis (3)
					Lack of fever (3)	Organism type (6)
					Intact mental status (2)	Elderly/frail patient [likely to colonized candida] (3)
					No urinary symptoms (2)	
**Total**				21 (53%)		Leukocytosis, pyuria, frailty cited by 3-7 respondents in every case

*CA-ABU=* catheter associated asymptomatic bacteriuria, *CA-UTI* catheter associated urinary tract infection.

#### Analyses for phase 1

First, we categorized each of the signs or symptoms participants identified as influencing their decision as (1) a guidelines-concordant clinical cue for distinguishing between CA-UTI and CA-ABU, or (2) a guidelines-discordant clinical cue that should not be used to distinguish between CA-UTI and CA-ABU. We then calculated Fleiss’ kappa to examine the overall inter-rater reliability of diagnoses for the four cases across all 10 clinicians, as well as the inter-rater reliability of clinicians that reported using the guidelines in their decision-making and those that reported not doing so. The MAGREE.SAS macro in SAS Version 9.2 was used to calculate the generalized kappa of Fleiss.

### Phase 2 – guideline-based algorithm development and validation

#### Algorithm development

**Figure 1:**
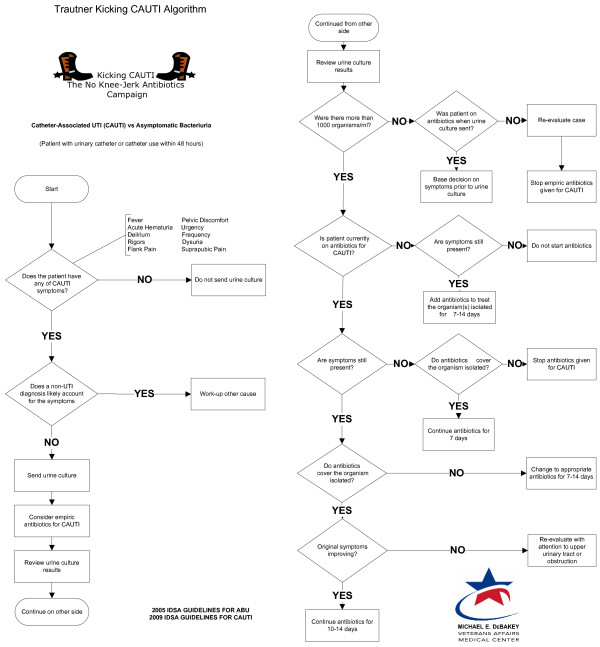
**Final form of the comprehensive algorithm.** This figure represents two sides, front and back, of a pocket card used in our guidelines implementation project, “Kicking CAUTI Campaign.”

We prepared a diagnostic algorithm for catheter-associated bacteriuria based on the IDSA guidelines. This evidence-based, diagnostic algorithm was designed to improve clinicians’ diagnostic ability to distinguish between CA-UTI and CA-ABU. The first version of the algorithm, developed according to the 2005 IDSA guidelines on CA-ABU and the 2009 IDSA guidelines on CA-UTI [1,2], was formatted as a flowchart to fit onto a pocket-sized card for high portability. The algorithm was evaluated for content and face validity, and revisions were made accordingly after each evaluation (see below for details). The final version of the algorithm was then used by three trained research personnel to classify cases of bacteriuria as either CA-UTI or CA-ABU (See Figure 1).

### Algorithm validation: content validity

To examine content validity of the algorithm, the initial version was distributed via email to all 11 expert panel members of the IDSA CA-UTI and CA-ABU guidelines committee. In a cover letter to our email message, we asked three survey questions (see Table 4 for specific wording); each question maps to one of the following standards: criterion, diagnostic, and feasibility. The response to each question was scored on a 10-point scale, with higher numbers indicating stronger agreement. We also provided a space for respondents to make comments or suggestions regarding the algorithm. Seven (64%) panel members responded with a numeric score, while one additional panel member provided comments only. The mean score for each question and each respondent was computed, and comments were tabulated for review by our research team.

**Table 4:** Ratings of the diagnostic algorithm by expert members of the Infectious Diseases Society of America (IDSA) guidelines panel for diagnosing and managing catheter associated bacteriuria

	**Content meets**	**Content meets**	**Content meets feasibility standard**
	**Criterion standard**	**Diagnostic standard**	
Questions posed to IDSA guidelines panel members	Does the algorithm appropriately reflect the definition of CA-UTI as per the IDSA guidelines?	Does the algorithm reflect an appropriate approach to diagnosis and treatment of CAUTI vs. ABU, as recommended by the IDSA CAUTI guidelines?	Could you apply this algorithm to your own catheterized inpatients?
Experts’ ratings, mean (standard deviation)	8.1 (*sd=1.1*)	7.1 (*sd=1.2*)	8.0 (*sd=1.6* )

(N=7).

Ratings were based on 10-point numerical scale with higher numbers indicating stronger agreement.

### Algorithm validation: face validity

#### Design and participants

We conducted cognitive interviews with six non-expert clinicians recruited from a purposive sample of clinicians working in local acute and extended care facilities to evaluate face validity of the algorithm. These participants, four internal medicine resident physicians, one nurse practitioner, and one physician assistant were chosen because all routinely provide care for catheterized adults and thus would be potential users of the algorithm.

#### Procedures

Participants were asked the following question regarding each step of the algorithm: “As you look at this diamond (decision point) or box (procedure step), what are you thinking it refers to?” Cognitive interview responses were categorized based on whether there was a misunderstanding, wrong interpretation, over-interpretation, correct interpretation, or off-topic response to each step of the algorithm [22]. The number of responses in each category was tallied, and percentage of total responses falling into each category was calculated. Based on the answers given, the algorithm was further revised to its ultimate form. This final version (see Figure 1) was sent back to the lead authors of the relevant IDSA guidelines [1,2] for their final input; neither suggested any substantial changes.

### Algorithm reliability: inter-rater reliability between non-experts and clinical expert

In order to determine if use of a guideline concordant algorithm has the potential to recalibrate inaccurately built mental models resulting in improved diagnostic accuracy of CA-UTI and CA-ABU, one expert and two non-expert providers used the algorithm to classify 71 distinct cases of catheter-associated bacteriuria arising in our local acute and extended care facilities as either CA-UTI or CA-ABU. Reliability ratings between non-expert and expert raters were used to confirm the ability of the algorithm to improve diagnostic accuracy. We also calculated inter-rater reliability of the algorithm between ratings of the two non-experts. Cases were chosen consecutively at 3-4 month intervals over a 10 month period. Each case was classified independently by the expert and at least one non-expert, resulting in 110 paired comparisons (as 20 cases were rated by the expert and both non-experts). Non-expert raters were trained to use the algorithm in introductory exercises prior to performing Cohen’s simple kappa the case classifications. Following procedures identical to those we used in Phase 1, the three raters were each given full access to the patients’ medical records including dates of the relevant urine cultures. Raters classified each case independently, and each rater was blinded to the other raters’ classifications. Because we were interested in the inter-rater reliability between specific pairs of raters, Cohen’s simple kappa was used to examine inter-rater reliability of accurate diagnoses aided by the algorithm between the expert and each non-expert and explore inter-rater reliability of the algorithm between non-experts [24].

## Results

### Phase 1– clinicians’ decision-making when diagnosing CA-UTI

Twenty-one (53%) of the 40 bacteriuria diagnoses by study participants were guideline-concordant (Table 3). Furthermore, only seven of the ten clinicians said that they applied the IDSA guidelines to arrive at their decisions; the other three said they did not use the guidelines by intention or they had not read the guidelines.

In terms of guideline-concordant clinical cues, six clinicians consistently identified fever as a guideline-concordant cue for CA-UTI (Table 3), and two clinicians commented on the presence or absence of urinary symptoms as being influential in their decision-making. On any given case, guideline-discordant cues (Table 1, right column) were endorsed by five or more clinicians when attempting to distinguish CA-ABU from CA-UTI.

All ten clinicians correctly identified case 4 as CA-ABU (Table 3), but some for the wrong reasons, as six reported that the organism type (*Candida*) influenced their decision, and three reported that the lack of leukocytosis influenced their decision. Interestingly, three clinicians also cited the presence of a chronic catheter in an elderly patient as a significant factor in their decision. This sign lead them to think that the patient was more likely to be colonized with a fungal agent. In Table 3 case 3, only one clinician correctly identified this case as a CA-ABU as the others were misled by guideline-discordant cues, e.g., leukocytosis and weakness in the patient. In Table 3 case 2, six clinicians correctly identified fever as a clinical cue for CA-UTI, but only four clinicians concluded that the patient had CA-UTI. Additionally, three clinicians each endorsed organism type and low number of organisms as leading them to conclude the patient had CA-ABU. In essence, clinicians could not distinguish which of these cues were guideline-concordant for CA-UTI. For Table 3 case 1, six clinicians arrived at the diagnosis of CA-ABU, but seven reported the guideline-discordant cues of leukocytosis as influential in their decision making. In this case, the patient was on oral steroids and therefore had an alternative explanation for his leukocytosis. The IDSA guidelines explicitly state that leukocytosis is not a reliable clinical cue for CA-UTI [1,2].

Inter-rater reliability among all 10 clinicians was fair (Fleiss’ kappa = 0.35, 95% CIs = 0.21 and 0.50) [24]. Inter-rater reliability among the seven clinicians reporting that they used the guidelines was also fair (Fleiss’ kappa = 0.28, 95% CIs = 0.07 and 0.50). Inter-rater reliability among the three clinicians reporting that they did not use the guidelines was substantial (Fleiss’ kappa = 0.63, 95% CIs = 0.06 and 1.00), i.e., they arrived at the same diagnosis, but these diagnoses were not always guideline-concordant [24]. Therefore, despite the higher reliability rating, these clinicians’ mental modes resulted in poorer diagnostic accuracy.

### Phase 2 – guideline-based algorithm development and validation

#### Content validity

Eight (73%) of the 11 members of the IDSA guidelines committee responded to our request for comments on the original algorithm. We received 27 specific comments addressing about half of the processes (boxes) or decision points (diamonds) in the algorithm. Ten (37%) of these overall comments concerned changing the recommended duration of treatment to reflect the patient’s response to therapy; we had misinterpreted this point in the guidelines. Seven of 11 members of the IDSA guidelines committee scored the algorithm along three standards (criterion, diagnostic, and feasibility) for measuring the quality of the algorithm content. Table 4 provides the mean ratings provided by IDSA guidelines committee members for each standard with each of the standards having an acceptable mean rating between 7.1 and 8.1. We modified the algorithm format to fit standard flow-charting in response to specific suggestions.

#### Face validity

Non-expert clinicians (see second portion of Table 2) reviewed the algorithm. Cognitive interviews with six clinicians produced comments for a total of 164 distinct comments about processes (boxes) or decision points (diamonds) contained within the algorithm. For 123 (75%) of the comments, respondents correctly interpreted the meaning of the process or decision point of the algorithm. Eighteen (11%) of the responses were incorrect, 13 (8%) were over-interpreted, 7 (4%) were misunderstood and 3 (2%) responses were off-topic. An example of over-interpretation was a box that stated “work up another cause,” which was interpreted as “do a chest x-ray and obtain an abdominal film.” The diamond that received the greatest number of incorrect responses was originally worded “Bacteriuria ≥10^3^ CFU/ml?”. We subsequently modified the text to read “Were there more than 1000 organisms/ml?” to reduce the need for real-time mathematical transformations and make interpretation unambiguous. The algorithm was modified to address the issues raised in the cognitive interviews, leading to its final form (see Figure 1), as approved by the two lead guidelines authors.

#### Reliability of the algorithm for diagnostic accuracy and inter-rater reliability

Using the final version of the algorithm, three providers classified 71 cases of catheter-associated bacteriuria. Of these cases, 28 were CA-UTI and 42 were CA-ABU as per the IDSA definitions for these conditions. Forty-nine cases were rated by both the expert and non-expert #1, forty-one were rated by both the expert and non-expert #2, and twenty cases were rated by both non-expert #1 and non-expert #2. Inter-rater reliability between the expert and non-expert #1 was substantial (Cohen’s kappa = 0.72, 95% CIs = 0.52 and 0.93). Inter-rater reliability between the expert and non-expert #2 was almost perfect (Cohen’s kappa = 0.80, 95% CIs = 0.61 and 0.99). The average inter-rater reliability among the expert and both non-experts was substantial (average Cohen’s kappa = 0.76), suggesting improved diagnostic accuracy among non-experts with the clinical expert (criterion standard). Inter-rater reliability between the two non-experts was also substantial (Cohen’s kappa = 0.88, 95% CIs = 0.64 and 1.00) [24].

## Discussion

Our data show that clinicians who routinely care for patients with urinary catheters use mental models that are often guidelines-discordant when classifying cases of catheter-associated bacteriuria as either CA-UTI or CA-ABU. Their decision cues consist of a heterogeneous group of signs and symptoms, many of which are not supported by evidence or run counter to evidence, as per IDSA guidelines [1,2]. The low level of accuracy and reliability of these clinicians’ diagnoses underscores the need for recalibrating their mental models to be compatible with evidence as documented in the IDSA guidelines for catheter-associated bacteriuria.

To address this need, we developed and validated an algorithm to enhance adoption of IDSA guidelines into diagnostic decisions for catheter-associated bacteriuria. A comprehensive version of the algorithm was created by mapping key decision points outlined in the CA-UTI and CA-ABU guidelines. Expert members of the IDSA guidelines panels provided content validation of the comprehensive algorithm with ratings along a 10-point scale for criterion, diagnostic, and feasibility standards. Cognitive interviews further established the face validity and usability of the comprehensive algorithm. From these results, we revised the algorithm. Finally, we established the reliability of the algorithm for accurately diagnosing cases as CA-UTI versus CA-ABU between expert and non-expert users and the inter-rater reliability of the algorithm between two non-expert users. High reliability between the clinical expert and each non-expert suggests improvement in diagnostic accuracy aided by the algorithm.

The results of the current study build on previous work that demonstrates physicians are more likely to treat bacteriuria with antibiotics (and therefore assume that the patient has a UTI) when patients have clinical cues that are consistent with prior diagnostic norms and practice (e.g., bacterial as opposed to fungal infection, higher white blood cell counts in the urine, positive urine nitrites, or a change in vague behaviors from baseline) [6,12,23]. The use of such guidelines-discordant cues leads to the inappropriate antimicrobial treatment of CA-ABU, and, as seen in our study, inconsistent (i.e., poor inter-rater reliability) and inaccurate diagnostic decision-making among clinicians. These guideline-discordant signs and symptoms are present within mental models that clinicians use to make diagnostic and treatment decisions [25].

Valid mental models built on prototypical cues (guideline-concordant signs or symptoms in Table 1) for CA-UTI can help to differentiate CA-UTI from CA-ABU among patients with catheter-associated bacteriuria. However, when these mental models are incorrectly constructed using cues that do not have high predictive validity (e.g., pyuria, and other guideline-discordant symptoms) or cannot help to differentiate the two subgroups (e.g., bacteriuria is present in both CA-UTI and CA-ABU), poor diagnostic accuracy and reliability will be the result.

The diagnosis and management of catheter-associated bacteriuria can be improved through the recalibration of clinicians’ mental models so that they are concordant with IDSA guidelines for differentiating CA-UTI from CA-ABU. This recalibration requires mindfulness of the guideline-discordant cues clinicians use when making diagnostic errors and substitution of guideline-concordant cues. Our study is consistent with prior evidence suggesting that simple methods, such as the use of checklists, algorithms, or protocols, combined with interventions such as audit and feedback, can enhance guideline adoption [26-28].

The current study has several limitations. Participants in our sample are not representative of clinicians from all fields of medicine, but they do reflect a group of clinicians (physicians and allied health providers) who regularly manage catheter-associated bacteriuria in a typical inpatient setting. Another limitation is that the two non-experts were trained by an infectious disease expert who was very familiar with the guidelines content and how to use the algorithm. However, this training process is reflective of how algorithms are often implemented in real-world setting. Indeed, we are currently studying case-based audit and feedback as a focused training method for using our algorithm to decrease inappropriate use of antibiotics for CA-ABU [28]. Another limitation is the modest sample size in Phase 1; however, the number of case pairs in Phase 1 was sufficient for reliability testing. Finally, the 10 cases were chosen to be “difficult” in that each challenged clinical norms, thus likely magnifying the disagreement between the clinician’s diagnosis and the guidelines-concordant diagnoses. Case classification, albeit time-consuming, is a fundamental first step for any quality improvement project related to CA-UTI. The algorithm developed in this study may improve the efficiency and reliability of case classification.

## Conclusions

During the diagnostic process, clinicians commonly compare patient’s symptoms to previously constructed mental models associating signs and symptoms to diseases. We have shown that use of improperly constructed (guideline discordant) mental models may result in diagnostic errors. Guidelines serve many of the same functions as mental models, in that they help identify data that are relevant to a particular diagnosis and exclude irrelevant data. The length and complexity of many guidelines limit their feasible dissemination and adoption in busy clinical settings. We have also shown that algorithms that simplify guidelines to better support decision-making in medical settings may help physicians identify and recalibrate inaccurate mental models, move toward more evidence-concordant diagnostic decisions, and reduce diagnostic errors.

## Competing interests

The authors declare that they have no competing interests.

## Authors’ contributions

BT conceived of the study, participated in its design, drafted the manuscript and had final approval of the version submitted to the journal. RB participated in study design, obtained data and contributed to interpretation, and helped draft the manuscript. AA conducted statistical analysis and data interpretation, and helped draft the manuscript. SH participated in study design, contributed to interpretation of data, and revised manuscript critically. AG obtained data and contributed to interpretation. PK participated in study design, contributed to interpretation of data, and revised manuscript critically. VP contributed to interpretation of data and manuscript revision. AN participated in study conception and design, helped draft the manuscript, and had final approval of the version submitted to the journal. All authors read and approved the final manuscript.

## Pre-publication history

The pre-publication history for this paper can be accessed here:

http://www.biomedcentral.com/1472-6947/13/48/prepub
